# Supplementary material for: Non-conventional mechanism of ferroelectric fatigue via cation migration
Source: Nat Commun. 2019 Jul 11;10:3064. doi: 10.1038/s41467-019-11089-w (PMC6624312; doi:10.1038/s41467-019-11089-w)
Supplement: Supplementary file 1 — Supplementary Information [file 41467_2019_11089_MOESM1_ESM.pdf]

Supplementary Materials

Non-conventional Mechanism of Ferroelectric Fatigue via Cation Migration

Anton V. Ievlev[1,*], Santosh KC[2], Rama Vasudvan[1], Yunseok Kim[3], Xiaoli Lu[4], Marin Alexe[5], Valentino R.Cooper[2], Sergei V. Kalinin[1], and Olga S. Ovchinnikova[1,*]

[1]The Center for Nanophase Materials Sciences, Oak Ridge National Laboratory, 1 Bethel Valley Rd., Oak Ridge, TN 37831

[2]Materials Science and Technology Division, Oak Ridge National Laboratory, 1 Bethel Valley Rd., Oak Ridge, TN 37831

[3]School of Advanced Materials Science and Engineering, Sungkyunkwan University, 2066 Seobu-ro, Jangan-gu, Suwon, Gyeonggi-do 16419, Republic of Korea

[4]The State Key Discipline Laboratory of Wide Band Gap Semiconductor Technology, Xidian University, Xi'an, 710071 Shaanxi, China

[5]Department of Physics, University of Warwick, Coventry CV4 7AL, UK

*Author to whom correspondence should be addressed. Email: ievlevav@ornl.gov, ovchinnikovaos@ornl.gov

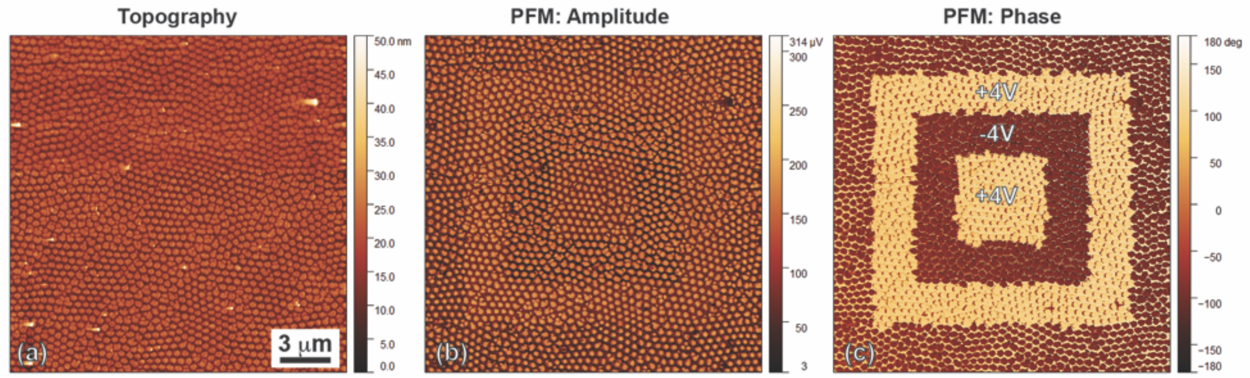

Supplementary figure 1. Local switching of PZT film with sub-micron-sized copper electrodes. Switching in 3 nested squares with sizes of 15, 10 and 5 μm and applied biases of +4, -4 and +4V, respectevily. (a) AFM topography, (b) PFM amplitude and (c) PFM phase of resulted structure.

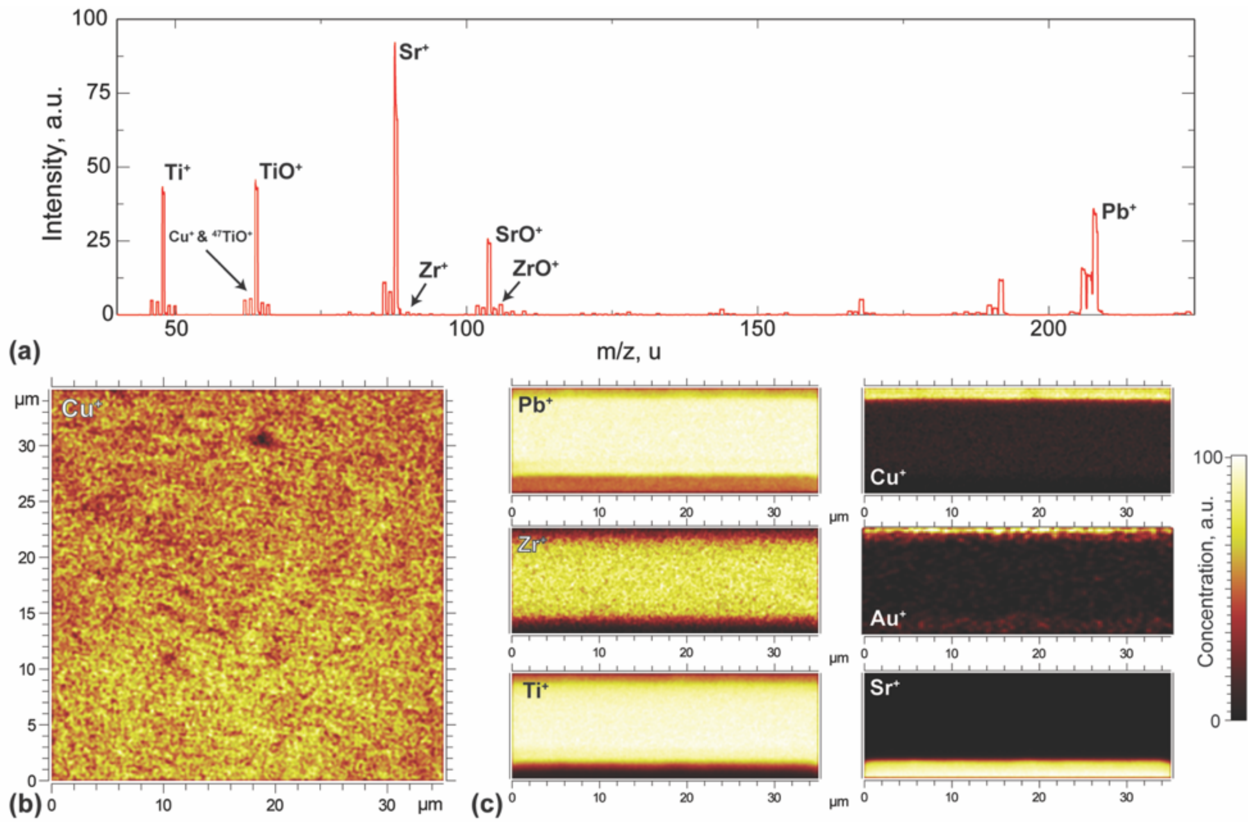

Supplementary figure 2. SIMS imaging of the pristine PZT sample. (a) Mass spectra averaged over the whole dataset, including Au/Cu top electrodes, PZT film and $SrRuO_3$ buffer layer; (b) XY map of copper on the sample surface; (c) XZ cross-sections of base elements Pb^+, Zr^+, Ti^+, Cu^+, Au^+ and Sr^+ as labeled.

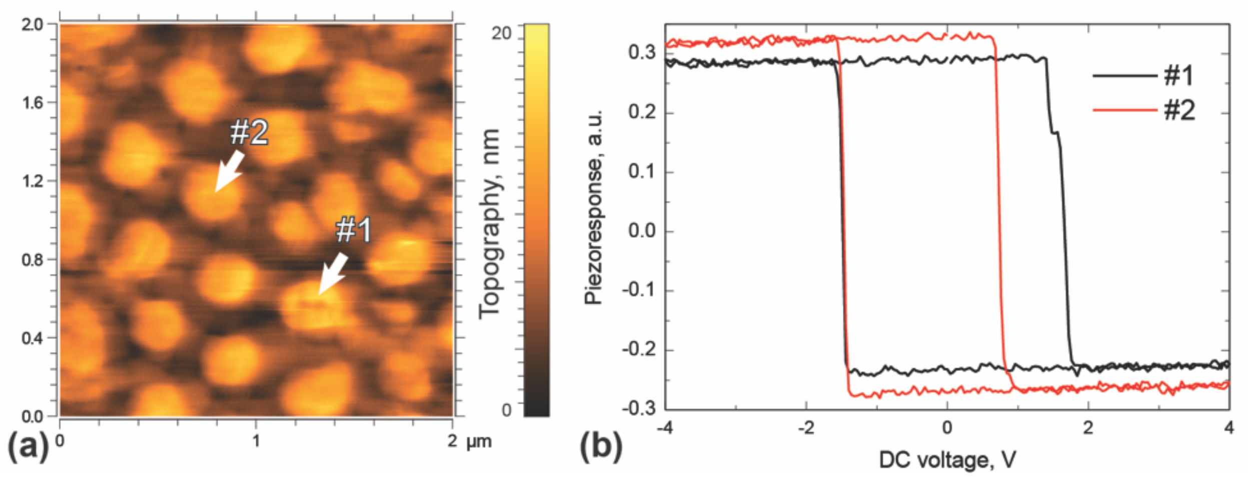

Supplementary figure 3. (a) AFM topography image of top Au/Cu electrodes; (b) First local hysteresis loops measured in two capacitors using the same AFM tip. Measurements in capacitor #2 were obtained after capacitor #1 was cycled with 10^7 switching pulses using same tip.

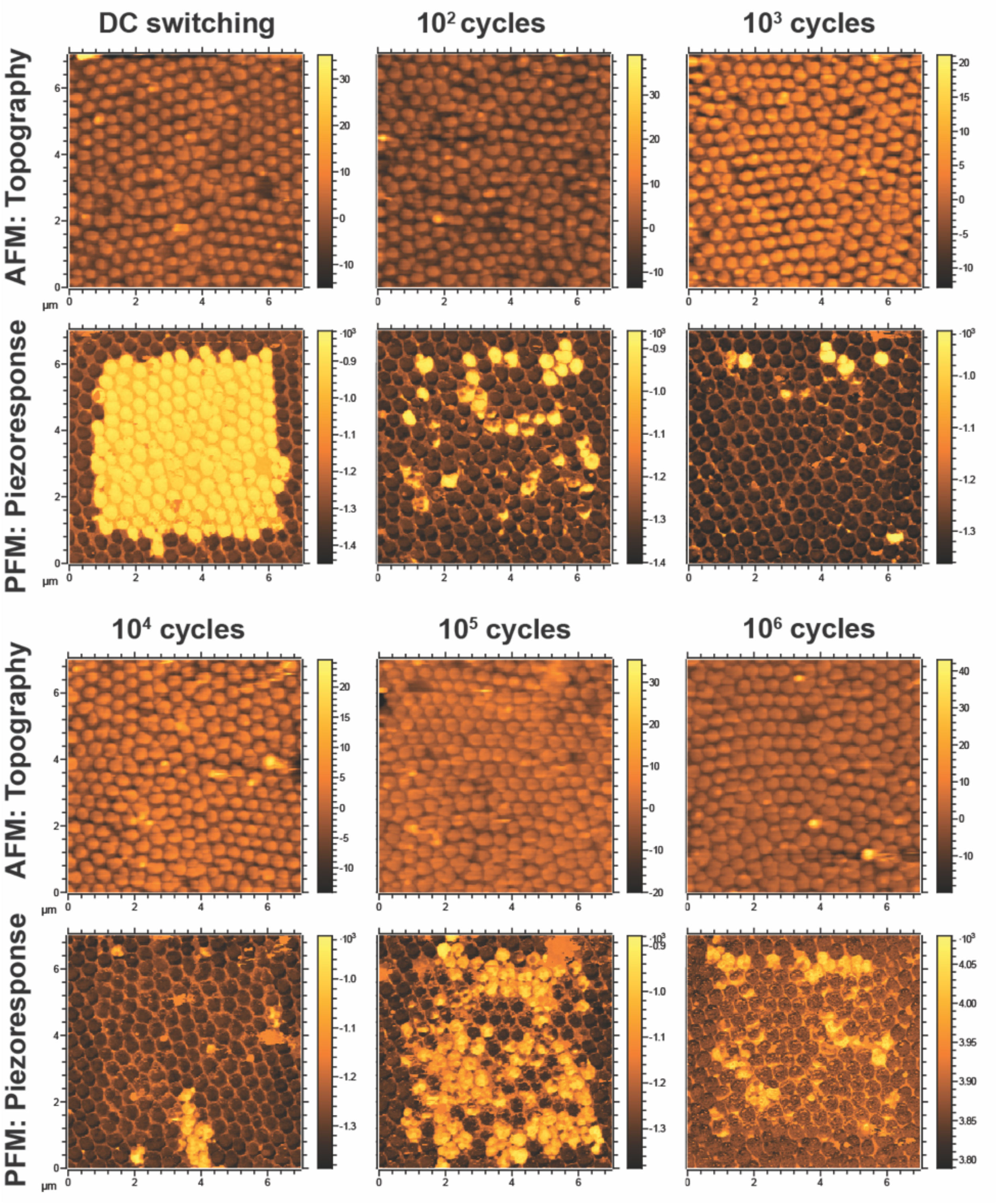

Supplementary figure 4. Detailed PFM imaging of the 5x5 mm^2 areas cycled by different numbers of switching pulses as labeled. Signals of the AFM topography and PFM combined piezoresponse.

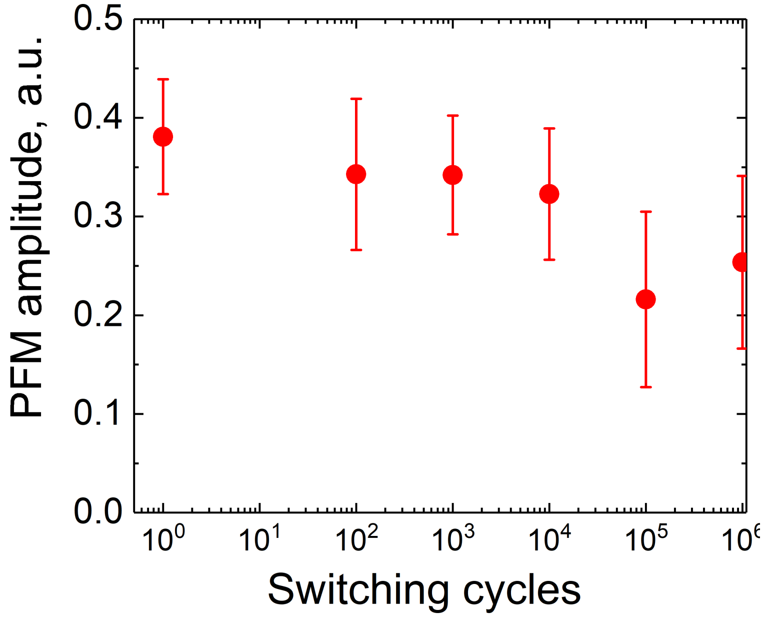

Supplementary figure 5. Signal of PFM amplitude averaged over cycled regions as a function of the number of switching cycles. Error bars calculated as standard deviation of all values from averaged area (5000 pixels).

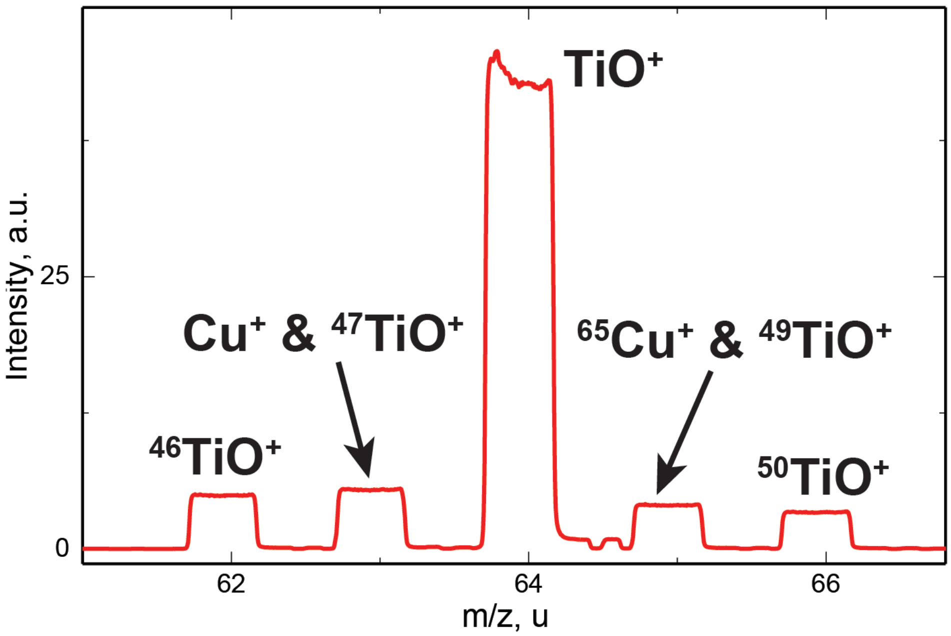

Supplementary figure 6. Mass spectrum averaged over electrode and PZT film medium zoomed in on the TiO^+ and Cu^+ peaks with isotopes.

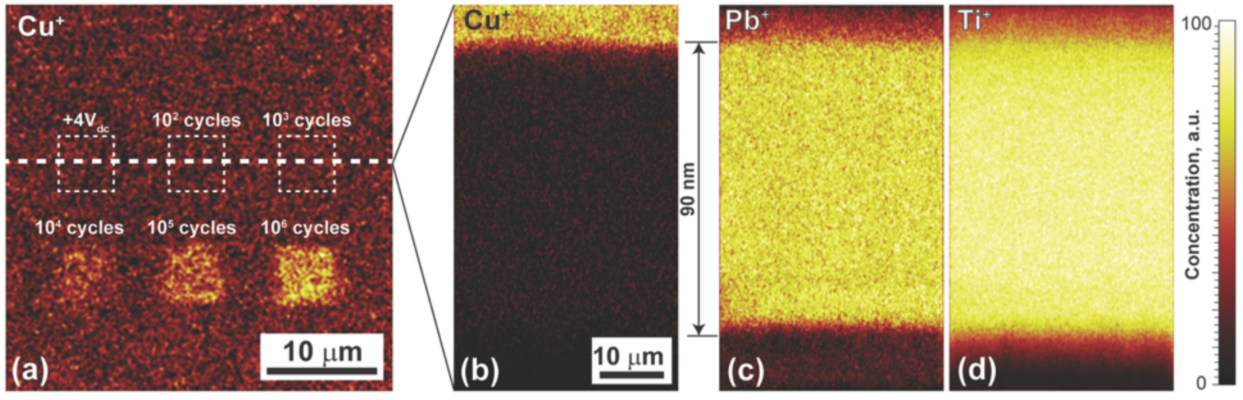

Supplementary figure 7. ToF-SIMS imaging of regions switched by DC voltage and cycled by 10^2 and 10^3 switching pulses. a) XY map of Cu^+ distribution averaged over 5 nm surface layer of PZT right underneath the electrode; (b,c) XZ cross-sections of (b) Cu^+ and (c) Pb^+ averaged over the area of regions switched by dc voltage and cycled with10^2 and 10^3 pulses.

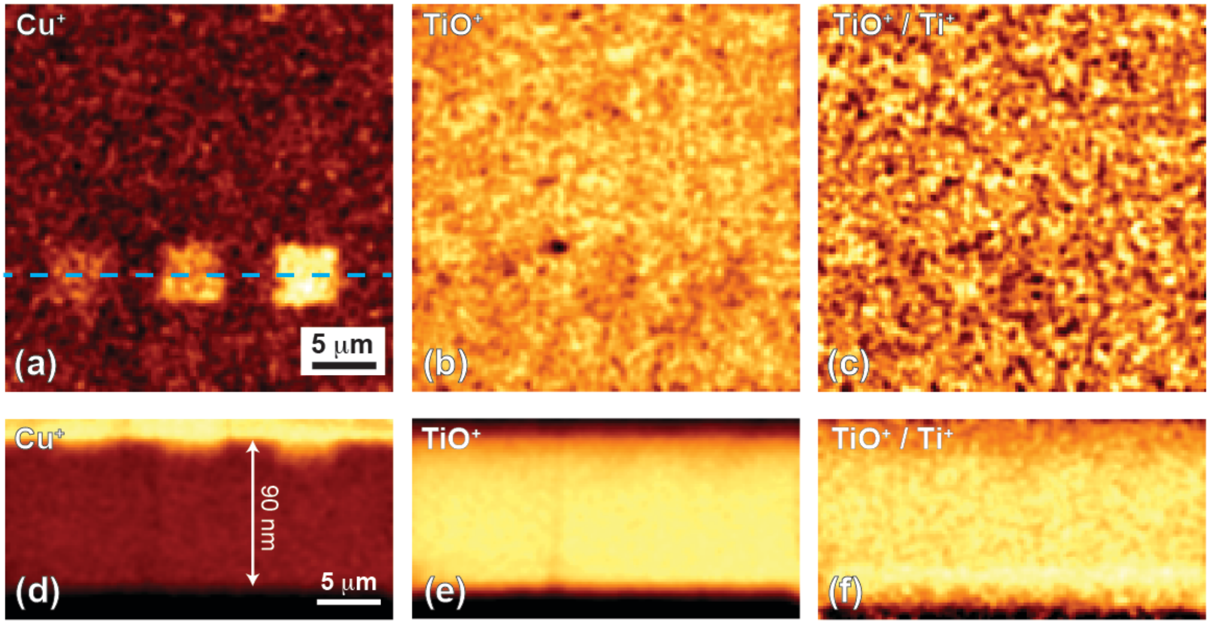

Supplementary figure 8. ToF-SIMS images of cycled regions. (a-c) XY maps and (d-f) XZ cross-sections over the region labeled in (a). Signals of (a,d) Cu^+ , (b,e) TiO^+ and (c,f) TiO^+/Ti^+ ratio.

Supplementary table 1. Results of DFT simulations of $Pb_{1-x}Cu_xZr_{0.125}Ti_{0.875}O_3$ for x = 0 to 0.50.

Fraction Cu	a [Å]	b [Å]	c [Å]	c/a	Total P [C/m^2]	Ave. Cu Z*	Ave. Pb Z*	Ave. Zr Z*	Ave. Ti Z*	E_{gap} eV
0	3.895	3.895	4.069	1.045	0.830		3.683	6.352	6.061	2.077
0.125	3.890	3.891	4.082	1.049	0.782	1.267	3.702	6.256	5.885	1.018
0.167	3.891	3.892	4.075	1.047	0.752	1.302	3.721	6.262	5.937	0.917
0.250	3.889	3.900	4.073	1.046	0.723	1.174	3.759	5.562	5.858	0.613
0.375	3.891	3.895	4.082	1.049						0.000
0.500	3.890	3.898	4.059	1.042						0.000
