# Supplementary material for: Non-conventional mechanism of ferroelectric fatigue via cation migration
Source: Nat Commun. 2019 Jul 11;10:3064. doi: 10.1038/s41467-019-11089-w (PMC6624312; doi:10.1038/s41467-019-11089-w)
Supplement: Supplementary file 3 — Source Data [file 41467_2019_11089_MOESM3_ESM.pdf]

```
x=0
   3.73655000000000
     2.9473928727147896    0.0002112971952252   -0.0000381359985367
     0.0002118911925515    2.9475623497191408   -0.0001529227838693
     0.0000631647581006   -0.0001011528844367    3.2691416996674612
   Pb   Zr   Ti   O
    24     3    21    72
Direct
  0.0038119066535925  0.9995031003470992  0.9502817868268919
  0.0065950272933480  0.0006215466863619  0.2870154270546955
  0.5005620743300275  0.4983836263826683  0.9499467203645569
  0.4989558258154690  0.4926211149997175  0.2905678987658351
  0.2491078784795985  0.7516025899342161  0.9506927407840300
  0.2417279227816135  0.7476367631465710  0.2870260519274995
  0.7501153496905335  0.2522160455632024  0.9498381196390032
  0.7512725653592909  0.2573790865863717  0.2905300502972392
  0.4976984630199756  0.0007607494860521  0.9484728529068913
  0.4955180791090467  0.0080651875084082  0.2886544249969328
  0.9993670965450276  0.5001162749297697  0.9523134769647097
  0.0002746148253424  0.4993834187726027  0.2886392130106126
  0.7506358215768707  0.7486680015326413  0.9479024891830177
  0.7574179382017641  0.7426590766808170  0.2917976545150811
  0.2500878231014791  0.2484117663314553  0.9527857688421412
  0.2492407993829266  0.2509744250229312  0.2857716982991044
  0.9915187498938364  0.0019944582343087  0.6226640729863169
  0.4998673050631389  0.5015321200658069  0.6219103877160143
  0.2524986354588299  0.7422084700466777  0.6227712079026669
  0.7505125228354668  0.2488974105027069  0.6222492090233511
  0.5091894446234547  0.9973829856672912  0.6224547595704439
  0.0002122068075854  0.4999388174281632  0.6223479678196457
  0.7486212327717906  0.7513568820261202  0.6215696229450952
  0.2497193793150813  0.2578154262924767  0.6228944250400991
  0.4983504080793130  0.7466970844199825  0.1352655614711271
  0.7525787553532828  0.0025397389801762  0.1345567999348819
  0.2499979249526652  0.0000584531914304  0.4702963589411359
  0.5019066734550210  0.2525150487016856  0.4685220361634838
  0.2500295244407615  0.0001933107349728  0.7995690828015067
  0.4981400289969545  0.2530489728436691  0.1307354656121901
  0.2483962395475427  0.0019718712405258  0.1326513446728436
  0.7505968277805223  0.0006310248972920  0.4673205812003637
  0.9982971502452792  0.2529901509742761  0.4677880821857894
  0.2495094037482792  0.4996412682732626  0.4669795028798719
  0.5000970928362849  0.7496335585962366  0.8003207648725924
  0.0000993041572136  0.7494787492898071  0.7983245033183657
  0.0019638251851518  0.2526856837289284  0.1303100338722578
  0.2474144745356052  0.4974966270238798  0.1303080791860693
  0.4998691756881275  0.2501965087205866  0.7983640276181615
  0.7524218078604856  0.4976494857706212  0.1314078916433189
  0.0014069525340337  0.7471991464335848  0.1301122062928494
  0.7504298676125806  0.0002542101577217  0.8017466671068433
  0.0001530287954240  0.2503695668072012  0.7997517263349314
  0.5018984467073904  0.7469084801759882  0.4676890178313887
  0.2497979045495102  0.4998750862083858  0.8010536439986506
  0.7504983057709329  0.4996196281473038  0.4687941894265349
  0.9983448381357544  0.7475717705878590  0.4686850208414322
  0.7502445940641900  0.4999692247790537  0.7987416546697311
  0.3736028824067465  0.1279925456568942  0.1542217209208076
  0.8822376726299339  0.1325464529430293  0.1617067297693669
  0.3672218488526763  0.6171533832916596  0.1611687200827680
  0.8773452810934071  0.6224981444063227  0.1560092436938717
  0.3802720131035351  0.1318031520464331  0.4935974839142613
  0.8729016911672282  0.1275448051081085  0.4925334781169817
  0.3746355845773108  0.6231970416712069  0.4921543442030955
  0.8729639402886978  0.6250941693113273  0.4915596894849170
  0.3728303283667830  0.1272910612812614  0.8226696265914463
  0.8739894657629143  0.1271608327973208  0.8202171584723723
```

| | | |
|---|---|---|
| 0.3725513829846857 | 0.6261932058114107 | 0.8202604207309099 |
| 0.8735186160242026 | 0.6259806993247369 | 0.8233969587697241 |
| 0.1240342668455843 | 0.3759829401688699 | 0.1569371880916231 |
| 0.6242211805211063 | 0.3761204677816012 | 0.1569222057223247 |
| 0.1234571537549579 | 0.8741753193331564 | 0.1537568262798188 |
| 0.6236783217665736 | 0.8767753354414962 | 0.1704479396089607 |
| 0.1250198244671697 | 0.3760255469786172 | 0.4936896417040148 |
| 0.6270253159819242 | 0.3751884419299619 | 0.4914255282143516 |
| 0.1198644925931164 | 0.8680677901361664 | 0.4937495233997808 |
| 0.6272393200001213 | 0.8732606974188571 | 0.4904112248716991 |
| 0.1273436691347487 | 0.3726724256385524 | 0.8224382571986285 |
| 0.6269222939132095 | 0.3733886875819992 | 0.8234853582220926 |
| 0.1268938188049492 | 0.8727311010329167 | 0.8226549564205784 |
| 0.6267685066367249 | 0.8732671457018384 | 0.8167629053941025 |
| 0.3726047109951511 | 0.3747497216669671 | 0.1567351442813537 |
| 0.8761074658569633 | 0.3750171380651657 | 0.1569075075480181 |
| 0.3689851490682520 | 0.8800635676576349 | 0.1571726668211713 |
| 0.8806174792028364 | 0.8680470146614137 | 0.1612053934431122 |
| 0.3759746466488479 | 0.3771770389284800 | 0.4918722849579791 |
| 0.8750088088874387 | 0.3769109023622466 | 0.4931077387524296 |
| 0.3815828703757190 | 0.8693316448664878 | 0.4935735561638385 |
| 0.8746600601506593 | 0.8752349717534713 | 0.4905525936028017 |
| 0.3771361605443990 | 0.3771242192528565 | 0.8226730165762416 |
| 0.8764104172584518 | 0.3764785753355567 | 0.8235043691248887 |
| 0.3761119725593469 | 0.8772389630251795 | 0.8202369727511618 |
| 0.8775969783622362 | 0.8757145810033973 | 0.8195699131187248 |
| 0.1240155258467328 | 0.1279269630631952 | 0.1537357412729724 |
| 0.6187890228376413 | 0.1321210457246647 | 0.1615151009184249 |
| 0.1249708991974921 | 0.6236194617524112 | 0.1568372303335719 |
| 0.6326932954433420 | 0.6189865589605397 | 0.1616003083891685 |
| 0.1178229251428183 | 0.1303876909543347 | 0.4944561863107967 |
| 0.6249850600738243 | 0.1249893245985641 | 0.4902152274315265 |
| 0.1241889933738513 | 0.6228681817409992 | 0.4917780970431008 |
| 0.6256750635350986 | 0.6226069824227699 | 0.4911315436009306 |
| 0.1226972019731408 | 0.1226842540259924 | 0.8226732835431347 |
| 0.6220741093504274 | 0.1240440210968716 | 0.8197269204038239 |
| 0.1231147672075216 | 0.6229054301923476 | 0.8227160783255937 |
| 0.6248334535678662 | 0.6226542710025161 | 0.8211694284909293 |
| 0.2494758028814064 | 0.0002554798976467 | 0.9833016899777427 |
| 0.7489866633215762 | 0.9992032301135617 | 0.9727131632891038 |
| 0.2496362263672478 | 0.4996551426455912 | 0.9834757772149322 |
| 0.7502428940522352 | 0.4991008792091124 | 0.9846689904288159 |
| 0.2457595109599994 | 0.0033258110749960 | 0.3075536240709383 |
| 0.7534205837302583 | 0.0041932236965847 | 0.3217618374295677 |
| 0.2471733375272424 | 0.4974485714410961 | 0.3212001699484782 |
| 0.7532784641157535 | 0.4971212915559962 | 0.3216280452042925 |
| 0.2496905786229455 | 0.0002765462152389 | 0.6538893968773600 |
| 0.7503653032206327 | 0.0010983318725564 | 0.6522128419675586 |
| 0.2497410046093041 | 0.4997267474554491 | 0.6539210068530106 |
| 0.7504094832047706 | 0.4995993660303020 | 0.6531636345002703 |
| 0.9998415408617016 | 0.2505401155678737 | 0.9833856993843690 |
| 0.5000394996274351 | 0.2511551403549461 | 0.9840787860884578 |
| 0.9998961302119719 | 0.7494551845326519 | 0.9834116395752156 |
| 0.5011421833181029 | 0.7500465269249037 | 0.9730655924532062 |
| 0.0015743849879409 | 0.2538471354189666 | 0.3216869582428014 |
| 0.4983308195328983 | 0.2540238465564650 | 0.3213250906227771 |
| 0.0018583221134498 | 0.7462911238988017 | 0.3214666271534833 |
| 0.4971043965649191 | 0.7451219511538756 | 0.3218816856983743 |
| 0.9987380512072084 | 0.2522189718144243 | 0.6538048619998796 |
| 0.5010137965580570 | 0.2528003327988436 | 0.6527673576271642 |
| 0.9987538565019402 | 0.7464249747931413 | 0.6527150318651779 |
| 0.5013605727953074 | 0.7467526972103720 | 0.6520847881846232 |

x=0.125

```
   3.73655000000000
     2.9447663945254354    0.0004440839889215    0.0061431228200513
     0.0004448841180067    2.9454091460288438   -0.0014870957760529
     0.0074920841807392   -0.0015383980346470    3.2773199851100152
   Cu   Pb   Zr   Ti   O
     3    21     3    21    72
Direct
  0.9990010414253067  0.9994177637060544  0.8502414722619530
  0.5015925990996781  0.4931487923366388  0.1850463858576974
  0.5069860711362421  0.0001598051233106  0.5194870583086583
  0.0058860324199706  0.0025300381616070  0.2886900442566885
  0.4996735956258848  0.4997553675495992  0.9464569659809609
  0.2427979806558660  0.7559144094603113  0.9561324806681968
  0.2405625739416984  0.7465919669882151  0.2932509614778148
  0.7481719091143073  0.2518409959410894  0.9538464997536856
  0.7436127267930647  0.2599541608555707  0.2945588153596282
  0.4950201981728337  0.0001511347607085  0.9527374452922458
  0.4924086659532727  0.0067512169145714  0.2831634883882831
  0.9976629101913477  0.4999774765072485  0.9572529361571571
  0.9991702589106568  0.4988500048082141  0.2951684592989925
  0.7483784661554844  0.7530934932072135  0.9517017841581463
  0.7541010648200591  0.7445235668105432  0.2955675694136728
  0.2455241975834805  0.2451525991573710  0.9589466433645852
  0.2511476448104056  0.2532594391214287  0.2906736653381053
  0.9898482774815162  0.0016667590058459  0.6177859167487938
  0.4987973560272873  0.5025167113213262  0.6264012237884304
  0.2498561482596376  0.7428608449738414  0.6287228014220471
  0.7440682590405678  0.2462579070722837  0.6256722634357021
  0.9970929772673125  0.4995057658702142  0.6250646305469250
  0.7400087911876160  0.7580758726432010  0.6272389642850604
  0.2476479947680941  0.2582097364647754  0.6284286009962986
  0.4980747733107673  0.7485867109768622  0.1389997297884800
  0.7572370001327841  0.0026280368022351  0.1317222990039297
  0.2492101740176121  0.0004391553416670  0.4755989857983802
  0.4993764672995548  0.2615790594817679  0.4673163899818559
  0.2542291270818513  0.0010877846371516  0.8015314745240916
  0.4972102811371867  0.2478049524683659  0.1338445102230773
  0.2438424173416023  0.0010295589399250  0.1325396003604107
  0.7540826493293403  0.0010934976124577  0.4685245808475123
  0.9978943737332775  0.2558554640562001  0.4694774418722124
  0.2482900382980984  0.4999488755348451  0.4666090389178776
  0.4974668761735178  0.7506717805008428  0.8005018194514131
  0.9984965506348981  0.7460980862292556  0.8021454109083263
  0.0023115185520557  0.2507727372932584  0.1316100961206840
  0.2421680830227568  0.4984620566117201  0.1345047338937853
  0.4979346228184303  0.2502422246168508  0.7978731331473961
  0.7601758021687057  0.4985426230926172  0.1352942096431828
  0.0018869341432922  0.7498624260953651  0.1306636650202418
  0.7410140662594743  0.0012827863443511  0.8013587635687958
  0.9988370399473618  0.2532862945072764  0.8049793562216224
  0.4993994893114126  0.7397319106050458  0.4680742021651998
  0.2505604507287214  0.4998033342707818  0.8043240343152248
  0.7496406587801661  0.5004218619771971  0.4685441163765337
  0.9973300306446768  0.7447484763756635  0.4713765799517513
  0.7483390321849878  0.5001551015962682  0.8003305027371690
  0.3735756745960094  0.1281679228421832  0.1554989181401323
  0.8829245989303592  0.1334046109704714  0.1659014188222435
  0.3720141256985640  0.6124485607631055  0.1685825175879630
  0.8765740673246909  0.6251106715060836  0.1575198109507325
  0.3878794021093145  0.1271785435640213  0.5021184023280855
  0.8747780886841159  0.1270910040011202  0.4909863485819707
  0.3706357576322230  0.6269517501739070  0.4906375533688028
  0.8752203276920772  0.6224517925894968  0.4959163520672590
  0.3718322830981556  0.1282651528206597  0.8217314657775674
  0.8773717849277223  0.1225553489978977  0.8277476257090929
  0.3766384762244670  0.6222004052485345  0.8256027516669958
```

| | | |
|---|---|---|
| 0.8761999181092538 | 0.6231820818821450 | 0.8231435650957518 |
| 0.1277627221861380 | 0.3729442856112073 | 0.1585243468471960 |
| 0.6269502038710204 | 0.3733874508840430 | 0.1604656702927639 |
| 0.1280357138682515 | 0.8717152960054678 | 0.1569446477191084 |
| 0.6318870199618775 | 0.8710541985055421 | 0.1669362477182401 |
| 0.1234191018080998 | 0.3778431715782431 | 0.4986954865879394 |
| 0.6293180237578802 | 0.3728309369884186 | 0.4890890340296253 |
| 0.1219877522137732 | 0.8658668291100147 | 0.4929163258241712 |
| 0.6262628072543786 | 0.8761101658255320 | 0.4966675403213190 |
| 0.3751196530004143 | 0.3752100785394135 | 0.1612020119793492 |
| 0.8782978244048283 | 0.3754149357628854 | 0.1586559795362825 |
| 0.3721373711845211 | 0.8823808221135629 | 0.1570239938528089 |
| 0.8847847805500227 | 0.8700366851018139 | 0.1648615709790231 |
| 0.3704689747042574 | 0.3731524607142895 | 0.4904976894612520 |
| 0.8750688510114695 | 0.3770097402162444 | 0.4978360658416263 |
| 0.3871026850402198 | 0.8731081998969610 | 0.5047396356443739 |
| 0.8739563667424022 | 0.8731037971418534 | 0.4892690571202607 |
| 0.3765269892306790 | 0.3760628493540020 | 0.8277480973222285 |
| 0.8734697527308724 | 0.3733058420065576 | 0.8249886174978817 |
| 0.3708976543695930 | 0.8722577669767539 | 0.8201244965214859 |
| 0.8778153295325334 | 0.8755244023027589 | 0.8266721740859762 |
| 0.1263628190251808 | 0.1281870842738518 | 0.1575245739304642 |
| 0.6205096279306849 | 0.1293826238560315 | 0.1611954826898652 |
| 0.1271496969297407 | 0.6253880199071197 | 0.1577082595500536 |
| 0.6292965084512450 | 0.6130398060150184 | 0.1668698605524411 |
| 0.1211990235917775 | 0.1341604321136246 | 0.4938040313764883 |
| 0.6260130935411735 | 0.1245726010963383 | 0.4962136302739959 |
| 0.1239584556816756 | 0.6223258925111559 | 0.4979739212862783 |
| 0.6290096682234899 | 0.6269774542294533 | 0.4891526481944471 |
| 0.1219248634018155 | 0.1225121262795306 | 0.8318001000028744 |
| 0.6259736775914799 | 0.1289528946525626 | 0.8180428361244364 |
| 0.1290561278255040 | 0.6279410692833705 | 0.8224441075338844 |
| 0.6268052359944920 | 0.6226550494855083 | 0.8255779599069240 |
| 0.2587538340597197 | 0.0006662152562712 | 0.9862833859252563 |
| 0.7379879870810214 | 0.9964933942576134 | 0.9724724874198686 |
| 0.2487193283741493 | 0.4999049385973847 | 0.9885482029707644 |
| 0.7549842308378333 | 0.4982057803828460 | 0.9893574294317925 |
| 0.2504989173865675 | 0.0022689731240378 | 0.3127042411523300 |
| 0.7530412965260382 | 0.0019654133837772 | 0.3236468770136342 |
| 0.2363324348028751 | 0.4985221752022648 | 0.3230798313368002 |
| 0.7634812295670179 | 0.4967340966934564 | 0.3234181291659892 |
| 0.2385254643202322 | 0.0017711345378790 | 0.6580608375796893 |
| 0.7620958974922557 | 0.0001024218578324 | 0.6544756530024304 |
| 0.2559732873247134 | 0.4991739334118628 | 0.6575704140508860 |
| 0.7446706004923875 | 0.4982597287945278 | 0.6553787641805382 |
| 0.0012144715826956 | 0.2599127658239646 | 0.9867882064265993 |
| 0.5006938069849098 | 0.2488200408364456 | 0.9881988063887024 |
| 0.0017313133627478 | 0.7403491141590375 | 0.9862179550033896 |
| 0.5035730521082378 | 0.7533395660275208 | 0.9766363094554300 |
| 0.0029759784309186 | 0.2575789531727024 | 0.3243316616792664 |
| 0.4984504228531242 | 0.2450240540299642 | 0.3229860932643273 |
| 0.0038667991929742 | 0.7426561110457620 | 0.3246672964171420 |
| 0.4975684995700267 | 0.7566387107748063 | 0.3244276088597000 |
| 0.0000365071581811 | 0.2494825992318493 | 0.6594930751286042 |
| 0.5012787621889412 | 0.2656603844056192 | 0.6547127273050277 |
| 0.9996928661844660 | 0.7507497362493714 | 0.6567933997747150 |
| 0.5016403713403492 | 0.7333262224837497 | 0.6548963694816744 |

x=0.167

3.73655000000000

| | | |
|---|---|---|
| 2.9455423157938627 | -0.0001544211055793 | 0.0029319442119151 |
| -0.0001626060744987 | 2.9462750175806107 | -0.0036380664296496 |
| 0.0033173589138680 | -0.0046064804747584 | 3.2713282053925430 |

| Cu | Pb | Zr | Ti | O |
|---|---|---|---|---|
| 4 | 20 | 3 | 21 | 72 |

Direct

| | | |
|---|---|---|
| -0.0002978404969512 | -0.0004465573041675 | 0.8542755046702309 |
| 0.0095330536323082 | 0.9995877253474171 | 0.1642978945478459 |
| 0.5008323384876358 | 0.5005885026748831 | 0.8522796716083020 |
| 0.9992661765382554 | 0.5008504748870675 | 0.5220645993906824 |
| 0.4973417299085119 | 0.4914153108314860 | 0.2914524562830704 |
| 0.2410778262765873 | 0.7586024682091983 | 0.9602552118795481 |
| 0.2381605589129701 | 0.7517553205026876 | 0.2909926009547413 |
| 0.7468510470278366 | 0.2553091057571577 | 0.9578941232951097 |
| 0.7499405381413626 | 0.2569458621769378 | 0.2955044647925023 |
| 0.4963563476307599 | 0.0005651372231232 | 0.9601825652444457 |
| 0.4956810179069502 | 0.0070109907865300 | 0.2925050018152928 |
| 0.9979556137072402 | 0.5006377348825228 | 0.9589503557398195 |
| 0.0007741617964296 | 0.4993077620554949 | 0.2856182246848110 |
| 0.7489776032828892 | 0.7512579361328959 | 0.9554482710946847 |
| 0.7556721206016203 | 0.7435038012777747 | 0.2954537495452870 |
| 0.2465345105690981 | 0.2471268337323720 | 0.9617379510427724 |
| 0.2469269357008362 | 0.2494379491017427 | 0.2901963086793558 |
| 0.9881352704476031 | 0.0022833805458505 | 0.6186745332210515 |
| 0.4979281079098691 | 0.5025916944008503 | 0.6190284624804059 |
| 0.2455353284637618 | 0.7406533437888356 | 0.6293491135362520 |
| 0.7514701211052508 | 0.2503750148139405 | 0.6289951034306803 |
| 0.5053630100615969 | 0.9988393684902708 | 0.6260857499436292 |
| 0.7485017922309324 | 0.7510593264990791 | 0.6285977431951650 |
| 0.2405338305789634 | 0.2618798238417042 | 0.6320545998361864 |
| 0.4988116384057601 | 0.7431208447581914 | 0.1346791921719196 |
| 0.7519295920729582 | 0.0023167502351813 | 0.1378882071813780 |
| 0.2473041831429442 | 0.0007827919137227 | 0.4697778145263976 |
| 0.5003380283434051 | 0.2491086648857539 | 0.4729433701869592 |
| 0.2519919499611912 | 0.0015363092716496 | 0.8039611180344595 |
| 0.4990734624353854 | 0.2539473176144564 | 0.1302617985799979 |
| 0.2549485843582406 | 0.0026651593155670 | 0.1355922499170355 |
| 0.7483950531977797 | 0.0009038541275224 | 0.4676241323311947 |
| 0.9962860774606482 | 0.2476784248012344 | 0.4680438106385281 |
| 0.2531883383318441 | 0.4995300002032827 | 0.4717178324913640 |
| 0.4977886391382549 | 0.7524593292798460 | 0.8054489022284947 |
| 0.9985756071481312 | 0.7448914191166967 | 0.8006136882714751 |
| 0.0018666614680588 | 0.2570553189980585 | 0.1327710447433703 |
| 0.2512772481002102 | 0.4968408710761948 | 0.1302390277798969 |
| 0.4982148144062734 | 0.2492659937807411 | 0.8038422017714811 |
| 0.7491895279236475 | 0.4962168847124971 | 0.1317487822534719 |
| 0.0007869494452329 | 0.7412850090552773 | 0.1327429137682838 |
| 0.7456756105115406 | 0.0013155153202114 | 0.8079173625042934 |
| 0.9985939600787574 | 0.2546439507959556 | 0.8044132175112992 |
| 0.5005898932680219 | 0.7509426426918785 | 0.4706616791863328 |
| 0.2458617355242833 | 0.5008814694857124 | 0.8059772309373396 |
| 0.7459161205400535 | 0.4995356609702447 | 0.4740494006147051 |
| 0.9957654129649360 | 0.7535592425184915 | 0.4691818599491345 |
| 0.7538568463689113 | 0.5007119823361865 | 0.8002279496928607 |
| 0.3770947636924847 | 0.1279305157237778 | 0.1561850683839896 |
| 0.8884480712386384 | 0.1287116777811614 | 0.1622448975020370 |
| 0.3673511689662655 | 0.6169317216437996 | 0.1670952742785155 |
| 0.8760603897137081 | 0.6223104891729322 | 0.1586664570182681 |
| 0.3825725168896261 | 0.1275995810591451 | 0.4997688306412247 |
| 0.8713377031895437 | 0.1291303184777806 | 0.4907510706046415 |
| 0.3730110068833727 | 0.6247330559040153 | 0.4913877279564713 |
| 0.8769129624765694 | 0.6216202749646871 | 0.5008997941663873 |
| 0.3716447756745701 | 0.1286355201877243 | 0.8210650735735626 |
| 0.8786536500609844 | 0.1224194279145618 | 0.8344344792655157 |
| 0.3777435365537148 | 0.6224263470568493 | 0.8310784137102062 |
| 0.8713884106852808 | 0.6281967596078826 | 0.8211173192353970 |
| 0.1277940584793285 | 0.3744080459419138 | 0.1594010567450082 |
| 0.6275547741141253 | 0.3726248446396233 | 0.1592407339115505 |
| 0.1286906611032674 | 0.8714620188007888 | 0.1503583232375162 |
| 0.6291685815085651 | 0.8698048948210508 | 0.1700233659623187 |
| 0.1208270913234453 | 0.3793588403942498 | 0.5051561680790240 |

```
  0.6288449535896468  0.3724360193341181  0.4920520622152295
  0.1228639081230117  0.8639064396421791  0.4926850244400330
  0.6257593911369213  0.8751400341720690  0.4968600701388518
  0.1300816665130592  0.3700001482611488  0.8208716080819715
  0.6235942508019401  0.3775436040284035  0.8319098542464690
  0.1221224915652007  0.8773767670969034  0.8342842496169666
  0.6289246307819546  0.8720429266478267  0.8125504570337044
  0.3734493768107342  0.3730497695065105  0.1597621355121229
  0.8756676958961177  0.3744128779951722  0.1599960238548771
  0.3734577031560128  0.8792682564968918  0.1593962386804139
  0.8875538101176234  0.8715689852622008  0.1609931603147310
  0.3730029709823319  0.3740670454455431  0.4920175964955995
  0.8776271640120517  0.3786419765979729  0.5028791607007677
  0.3809620763692073  0.8712089203526894  0.5003055297781466
  0.8710690716796354  0.8721859505799839  0.4899398685645758
  0.3779119405320955  0.3778000352935461  0.8326388111698642
  0.8702671867351048  0.3710112823468159  0.8213592118645607
  0.3714259448588548  0.8736603410944419  0.8172218458792245
  0.8786844428278096  0.8765302595197055  0.8315487894872993
  0.1282165886229211  0.1284375121694586  0.1501993592667976
  0.6204365738281606  0.1318261640382618  0.1632563842580426
  0.1265496596551225  0.6223341515601390  0.1592154903402005
  0.6328889999162982  0.6168658632834826  0.1661277550255968
  0.1223984456571833  0.1366139571584907  0.4940400338671159
  0.6255511624698222  0.1245271834160751  0.4976336878945933
  0.1219794781648754  0.6211577007195606  0.5014140095562255
  0.6280846324102731  0.6257599266384146  0.4903417800947509
  0.1218766191577374  0.1219757781491978  0.8357591363691751
  0.6274358988870608  0.1295931129049435  0.8189210665108386
  0.1297024200760125  0.6299540365017205  0.8211959304219576
  0.6247714459429321  0.6220895683349698  0.8303261740656481
  0.2653057229543173 -0.0005919988135204  0.9873705929269685
  0.7344088739989941  0.9961657235951651  0.9770072622171291
  0.2398095471058760  0.4987279108628245  0.9859746044238547
  0.7612477159277452  0.4975364987558241  0.9871813502950179
  0.2556477369140093  0.0031526935580736  0.3088927716233444
  0.7432359829697790  0.0032328830018918  0.3239031302984474
  0.2446325531996043  0.4954322082976578  0.3270827008570617
  0.7555121880982668  0.4952043940294979  0.3274806998428350
  0.2417026952448487  0.0014483067778593  0.6584977597987383
  0.7595451976939009  0.0019602904773307  0.6580573645119778
  0.2625252178100692  0.4999145553806651  0.6603366574085500
  0.7388661779485647  0.5001447464263254  0.6565320429460135
  0.0004780311203083  0.2665420921707134  0.9879912582581123
  0.5020515854225170  0.2414736601148691  0.9857987245147131
  0.0009810225807762  0.7341142853961631  0.9878307100194291
  0.5053232818917724  0.7656686589301978  0.9747717544238913
  0.0028006721667002  0.2635521300695060  0.3243545756896181
  0.5000625911065429  0.2505146626921367  0.3260123518178508
  0.0020740737975220  0.7377265280078350  0.3240291563947376
  0.4994067771905766  0.7491386410322333  0.3252195086257418
  0.9983933933354924  0.2369053095735889  0.6607831930886556
  0.5014261511876709  0.2579911427657721  0.6580826299631657
  0.9982095662317594  0.7600972804189657  0.6574018987577910
  0.5016599192524854  0.7403232839174428  0.6570116930948207
```

x=0.25

```
   1.00000000000000
    10.9983773335032193   -0.0006740070616612   -0.0069452201301477
     0.0009611246128272   11.0297425465900272    0.0119872892040470
    -0.0072811339653690    0.0126605621537270   12.2203395383415465
   Cu   Pb   Zr   Ti   O
     6   18    3   21   72
Direct
```

| | | |
|---|---|---|
| 0.0015437578330032 | 0.9991478135227468 | 0.8508957999197984 |
| 0.0027430178994868 | 0.0013896581990545 | 0.1722722258163803 |
| 0.2503514691475956 | 0.7512044176987295 | 0.8535151909842070 |
| 0.7488670352175556 | 0.2544829793769837 | 0.1906186219369111 |
| 0.5000263049684426 | 0.9991284429374803 | 0.8502400690063429 |
| 0.4928570113609598 | 0.0109845098785050 | 0.1717561534422879 |
| 0.9996836448253487 | 0.4951767287946370 | 0.3028881600025906 |
| 0.2511406740323580 | 0.2336822679156360 | 0.9713409253798989 |
| 0.2497517940633074 | 0.2416643412532000 | 0.2991437848128165 |
| 0.9925308053330304 | 0.0001983324772670 | 0.6143300684015356 |
| 0.2536202059894753 | 0.7433618933845318 | 0.6178393454125475 |
| 0.7482012395278567 | 0.7522905351451773 | 0.6250019904863803 |
| 0.5004958386617310 | 0.4993514284140078 | 0.9624783635002898 |
| 0.5036265522054153 | 0.4933526531653741 | 0.3043822626811984 |
| 0.2355463339879010 | 0.7436050984272988 | 0.2954805935882597 |
| 0.7503140709782469 | 0.2473920721465546 | 0.9545960344049289 |
| 0.0023774762157757 | 0.4988511253223355 | 0.9638140216266757 |
| 0.7494161813210428 | 0.7500642951314680 | 0.9624738632314650 |
| 0.7617131841394510 | 0.7458069693777080 | 0.3072622681490647 |
| 0.4998621216533681 | 0.4995457324173839 | 0.6239712806876057 |
| 0.7506995392954070 | 0.2443576859113131 | 0.6212880677745438 |
| 0.5088560103533026 | 0.9974371859873654 | 0.6129524637531915 |
| 0.0018493816487333 | 0.4972654728739204 | 0.6249755395886153 |
| 0.2511426087622996 | 0.2512361934106978 | 0.6254560908021750 |
| 0.4935754148837457 | 0.7451089481301864 | 0.1374130335711181 |
| 0.7524157901391567 | 0.0000377880875747 | 0.1416942640333043 |
| 0.2501676798862114 | -0.0000106395497264 | 0.4639438398863499 |
| 0.5045577669652013 | 0.2491806842941506 | 0.4646534993463195 |
| 0.2502370631707536 | 0.0016066381623157 | 0.8134059294384915 |
| 0.4927269115148604 | 0.2617678950935274 | 0.1384529977547819 |
| 0.2428792491562185 | 0.9965110924142551 | 0.1341096315946450 |
| 0.7514460258208563 | 0.0017459428773441 | 0.4618359701668617 |
| 0.9970279936603226 | 0.2499280778300074 | 0.4653569124968944 |
| 0.2502139840883520 | 0.4959770123075808 | 0.4727958945363925 |
| 0.5048030596974635 | 0.7411865080521564 | 0.8097782712890587 |
| 0.9964790677220884 | 0.7412912287168431 | 0.8075877136288907 |
| 0.0054371450495830 | 0.2596736401518914 | 0.1388863581596886 |
| 0.2472776716098483 | 0.5005035889352722 | 0.1322022786964673 |
| 0.5011896642914718 | 0.2565749703081909 | 0.8060617440842407 |
| 0.7525058371482982 | 0.5062799351853976 | 0.1406129854039539 |
| 0.0032381983577617 | 0.7408115803605344 | 0.1298007134914406 |
| 0.7506575090222204 | 0.0002488359828298 | 0.8131076742011776 |
| 0.9996835303991934 | 0.2564070613604444 | 0.8066168868689854 |
| 0.5035965318502282 | 0.7477452436859382 | 0.4680171696283663 |
| 0.2503057034798815 | 0.4930061130916668 | 0.8080813994167904 |
| 0.7508282924278542 | 0.4963533686643246 | 0.4717158611354837 |
| 0.9974967164966666 | 0.7478304710459476 | 0.4725114456618469 |
| 0.7510332208900489 | 0.4971975717028743 | 0.8012127732758894 |
| 0.3737599663736233 | 0.1314475422642297 | 0.1524698017952921 |
| 0.8795675699604751 | 0.1360838174031700 | 0.1733277550446047 |
| 0.3626448546359566 | 0.6209157085069145 | 0.1661795551527798 |
| 0.8753402002385126 | 0.6254805259296070 | 0.1610987284169455 |
| 0.3779613134925546 | 0.1338803606498120 | 0.4984462114881439 |
| 0.8744743497308922 | 0.1262882656178899 | 0.4933025077049493 |
| 0.3745126755909708 | 0.6239490141782573 | 0.4950247349371051 |
| 0.8715151125071758 | 0.6267242141821880 | 0.4982440458324740 |
| 0.3797289122218247 | 0.1222726765866292 | 0.8340088395978739 |
| 0.8779900553001067 | 0.1245634212389592 | 0.8311683862636704 |
| 0.3710872697638620 | 0.6277467031024941 | 0.8304710316236127 |
| 0.8746151907836665 | 0.6223886414490784 | 0.8235761766221946 |
| 0.1238858310092957 | 0.3783238636021706 | 0.1588688604298964 |
| 0.6276002603458710 | 0.3763300100607425 | 0.1678231960212327 |
| 0.1224766455471745 | 0.8732727622802254 | 0.1560190054571640 |
| 0.6106668036386843 | 0.8869707402754924 | 0.1685289683497389 |
| 0.1261340242325475 | 0.3746394238306112 | 0.5001453633861984 |
| 0.6236244531634964 | 0.3770752540622738 | 0.4918663361847797 |

```
  0.1218993287076625  0.8673407578012822  0.4965642081747131
  0.6291443796320700  0.8718233015273767  0.4946029021831118
  0.1227429960169176  0.3772366722412660  0.8250725673159883
  0.6277012484931462  0.3737158163675751  0.8296669167435097
  0.1259122655739095  0.8728547182737906  0.8392060904519545
  0.6215737837234601  0.8778908819900966  0.8268100393515925
  0.3733506275477408  0.3801274423254021  0.1585798836986256
  0.8721238780102589  0.3745398008823788  0.1688673749581404
  0.3715995498217787  0.8868121735663426  0.1587149342215213
  0.8875800175811488  0.8729200253301856  0.1601534720818633
  0.3767584889209400  0.3769475148164445  0.4981822750795438
  0.8786980572630132  0.3785532535827292  0.4929631453691746
  0.3805839220107284  0.8686334652038140  0.4948830186863182
  0.8731738403991630  0.8729069500795245  0.4971463549186670
  0.3770945001170440  0.3765428732067808  0.8254633041582089
  0.8724836152604424  0.3727754860409925  0.8298527041053011
  0.3744664828228547  0.8735657337898690  0.8399991290636656
  0.8808452151811770  0.8768705443225777  0.8292130859602810
  0.1188119359791610  0.1271640334663694  0.1553999431950297
  0.6187572408250108  0.1360458171297788  0.1696001421214730
  0.1228320291980975  0.6224395426768650  0.1620542018612874
  0.6303316615635796  0.6207751816413702  0.1643128457794058
  0.1220423462760988  0.1327118113311385  0.5000760342321999
  0.6261821064245244  0.1245888564410944  0.4912030968091438
  0.1237998565063487  0.6224486729921198  0.4965990535791117
  0.6253143765842177  0.6231700428185368  0.4989383232891063
  0.1210947572573991  0.1228313308098939  0.8314095272671658
  0.6233041473057898  0.1251229341876431  0.8306652018427778
  0.1292732626618865  0.6279776087035664  0.8308912005457489
  0.6276323731955885  0.6215190930853383  0.8198576676231260
  0.2454068745343091  0.0150132306086386  0.9902686273865274
  0.7502254410094502  0.0000126992594595  0.9812988974391026
  0.2484498976099071  0.4926677500007693  0.9873816085709565
  0.7512180306749069  0.5026901489075951  0.9933025578496358
  0.2457698539289891  0.0116518965844982  0.3067524979911209
  0.7559735231009216  0.9866659561211405  0.3222663902935006
  0.2443512224510053  0.4984797289612463  0.3272729597765069
  0.7524674272825490  0.5116086807933267  0.3265069611258631
  0.2503880176756201  0.9995133945662673  0.6675917459836632
  0.7514113519577288  0.0035897544435772  0.6643653094913219
  0.2501127878755689  0.5009664474664974  0.6613345599185616
  0.7516411675293515  0.4928390726634033  0.6565522517704508
  0.9980117204218818  0.2631593848468305  0.9929777959571333
  0.5021214230157091  0.2644152576348415  0.9929047569596468
  0.9904665102260364  0.7343159325145354  0.9872460864936450
  0.5190575423987166  0.7344197007640413  0.9786413788080044
  0.0147373957571714  0.2626124720639414  0.3233097148004396
  0.4860325973080697  0.2625543877556136  0.3223000158269790
 -0.0004635171134325  0.7397007913165733  0.3270237628250876
  0.5016334063048501  0.7368031933361523  0.3243596866432552
  0.9952284783705201  0.2519005993345174  0.6620503358850370
  0.5046033156558293  0.2514448523874955  0.6616504789387427
  0.0011059966424960  0.7479259614015336  0.6623102305810844
  0.4983595938345873  0.7499030593536619  0.6623850215501703
```

```
x=0.375
   1.00000000000000
    11.0065920400295578    0.0037025892986565    0.0077997038834290
     0.0053733910041527   11.0153164372518386    0.0308912531818810
     0.0093313146167498    0.0337895977764605   12.2465231260260854
   Cu    Pb    Zr    Ti    O
     9    15     3    21    72
Direct
  0.0034193227270879  0.9987362544824553  0.8575061094428085
```

| | | |
|---|---|---|
| 0.0046598215170703 | 0.9991643330950134 | 0.1794349104298388 |
| 0.2503600463046652 | 0.7517729401812099 | 0.8576478175137555 |
| 0.7488884224255419 | 0.2569508117643111 | 0.2027859563986580 |
| 0.4988402158252945 | 0.9980565057602004 | 0.8552777058240386 |
| 0.4937450680590292 | 0.0108032330326191 | 0.1802787106707792 |
| 0.0040848317446350 | 0.4979281339911374 | 0.2002150673284765 |
| 0.2506733098399739 | 0.2468314882828492 | 0.8582451429756222 |
| 0.2458617201200525 | 0.2473948830062536 | 0.1814349639787768 |
| 0.9944477584997179 | 0.0039225070052864 | 0.6164545105232219 |
| 0.2536591459511444 | 0.7472016140786991 | 0.6186873016239417 |
| 0.7474737855818876 | 0.7521509766742132 | 0.6236219020614564 |
| 0.4911857401683569 | 0.4985380514518322 | 0.9691281122302191 |
| 0.4876188213618328 | 0.4900739067246931 | 0.3176346697781384 |
| 0.2267056963451086 | 0.7586849622363659 | 0.3062976788967555 |
| 0.7506449554849911 | 0.2509066704015848 | 0.9630836132667167 |
| 0.9951156478045032 | 0.4946731785301272 | 0.9623509520245157 |
| 0.7439149117342710 | 0.7440084172197552 | 0.9678877623574779 |
| 0.7645365245472746 | 0.7415011516047155 | 0.3145394399844814 |
| 0.4971341481488538 | 0.4982501736098660 | 0.6232408963189398 |
| 0.7516138585651528 | 0.2449902483445752 | 0.6192141962817483 |
| 0.5056821752333159 | 0.9994008019465666 | 0.6130422431238665 |
| 0.9991784291281430 | 0.4959244174978749 | 0.6184331176072004 |
| 0.2504636238011784 | 0.2539181094491796 | 0.6150157470007144 |
| 0.4959152775387368 | 0.7463219603645298 | 0.1414083678058789 |
| 0.7519413037590550 | 0.9977675157566449 | 0.1437506432881461 |
| 0.2480305985490023 | 0.0061342640824698 | 0.4586164107705379 |
| 0.5046691149748349 | 0.2488674333749872 | 0.4621881026535595 |
| 0.2510777991279783 | 0.9995200265360475 | 0.8200990513433823 |
| 0.4961581810701349 | 0.2590066456528268 | 0.1426753024097736 |
| 0.2454364787327099 | 0.9951160021793701 | 0.1291486052517718 |
| 0.7538128115979019 | 0.0030232811453424 | 0.4637672759293126 |
| 0.9979736904687304 | 0.2530382417423149 | 0.4576074829519599 |
| 0.2457972440433943 | 0.4977825169671515 | 0.4657667886524828 |
| 0.5069799548758103 | 0.7413162419072623 | 0.8104562449543220 |
| 0.9925091901729002 | 0.7417586362717739 | 0.8081105476184215 |
| 0.0016248446758963 | 0.2530010405840321 | 0.1491060926609518 |
| 0.2513413266211090 | 0.5039467886353248 | 0.1362855154198291 |
| 0.5067123721517556 | 0.2554219415845870 | 0.8107647117658451 |
| 0.7465715939104459 | 0.5052851081713055 | 0.1499354259870352 |
| 0.0030997334303181 | 0.7461612462745936 | 0.1325446164942019 |
| 0.7502736746589022 | 0.9977075663778748 | 0.8161147464955109 |
| 0.9942174374786512 | 0.2544587127222954 | 0.8107689885140265 |
| 0.5023014878441417 | 0.7480467658573855 | 0.4693576367592130 |
| 0.2480557275784742 | 0.4994119202039120 | 0.8127186624642238 |
| 0.7506765070559385 | 0.4954674092701310 | 0.4691452798036218 |
| 0.9985993115180944 | 0.7490774453744563 | 0.4731736850844422 |
| 0.7496073956700801 | 0.4984021586918435 | 0.8000597315799320 |
| 0.3729910005486500 | 0.1282497668993911 | 0.1604524551879362 |
| 0.8754873217733152 | 0.1353681523573229 | 0.1726208057905019 |
| 0.3810667165234204 | 0.6057363849418351 | 0.1566071536334036 |
| 0.8865541085116589 | 0.6180562802254022 | 0.1779768900298187 |
| 0.3807466141074092 | 0.1319484580233820 | 0.4970471129487111 |
| 0.8749327813341718 | 0.1273295560831936 | 0.4974284158895850 |
| 0.3755422286855052 | 0.6235348234454219 | 0.4996737122058394 |
| 0.8708695477140104 | 0.6284387815367568 | 0.4939378100788748 |
| 0.3779112262288253 | 0.1262560296373231 | 0.8427441585526443 |
| 0.8808365504381386 | 0.1218079561953004 | 0.8373503210754557 |
| 0.3706275676609834 | 0.6270142687217466 | 0.8351935228013579 |
| 0.8746733360828531 | 0.6205251114366378 | 0.8265012471352778 |
| 0.1248857294308183 | 0.3778116298932778 | 0.1716214920668410 |
| 0.6277579112606545 | 0.3783907810199830 | 0.1679507692633824 |
| 0.1235214883115314 | 0.8700476420065455 | 0.1488234908928042 |
| 0.6115926664702586 | 0.8878384303926871 | 0.1785075808539818 |
| 0.1237407426208812 | 0.3775526338118115 | 0.4967697029755572 |
| 0.6228486497253272 | 0.3773647678944969 | 0.4969018958721996 |
| 0.1237690253698509 | 0.8701992913391988 | 0.5026224399018212 |

```
  0.6280704175424391  0.8726950622011395  0.4958955426178187
  0.1278256373495837  0.3698470856274583  0.8360675099206285
  0.6282895336431726  0.3740512892068334  0.8304440858345044
  0.1236137696414749  0.8719601167594507  0.8397437608385756
  0.6218688418611956  0.8762049099146817  0.8274232678243564
  0.3647448270028527  0.3689245563307497  0.1640040681816060
  0.8776141124364982  0.3818105991997227  0.1830982567764995
  0.3777043096714917  0.8888950628898511  0.1580590410597615
  0.8849863290172117  0.8693009685318540  0.1631971217581162
  0.3777932472884527  0.3795850242165661  0.4950023805838006
  0.8750095409398908  0.3767672494262643  0.4937109760167607
  0.3780385241458758  0.8703118098183993  0.4955967803614294
  0.8741662461913872  0.8743609357075046  0.5005333575137806
  0.3717018877408693  0.3706274599328943  0.8363537857888664
  0.8729980746652360  0.3739870954539293  0.8303611915755205
  0.3767379451092138  0.8727674862428665  0.8378743342700365
  0.8818850126622839  0.8773415008649714  0.8326826807594243
  0.1237148474942425  0.1275370383549772  0.1543394668961534
  0.6171859644802296  0.1325891215069114  0.1742360701263667
  0.1224163159838974  0.6187635654224339  0.1672766043977989
  0.6520069492200733  0.6419816141546978  0.1572532996116625
  0.1193129325794103  0.1329930297501106  0.5029335169959436
  0.6269597942255346  0.1251840476176865  0.4943181411914623
  0.1280838799134465  0.6271638057321182  0.4978129900246939
  0.6244289342071618  0.6243212344812648  0.5009650780327722
  0.1232469691946792  0.1268787354618623  0.8419710559255702
  0.6202442140729155  0.1216826964447922  0.8357327908496669
  0.1291801949001421  0.6292984525054813  0.8346478381366624
  0.6279375725783910  0.6205335845735885  0.8230736600018193
  0.2494888675331471  0.9996980617114379  0.9888163288968480
  0.7473489076007854  0.9954604893254151  0.9839795546713143
  0.2484173469277718  0.4976306330702264  0.9930715203364373
  0.7592605756342373  0.4988489831022938  0.0017631439201035
  0.2346765314352978  0.9847841635383973  0.3049122837213507
  0.7616624493804167  0.9899520529009166  0.3248572547338691
  0.2584884603883766  0.5085541307924399  0.3243871162845295
  0.7365548510055966  0.5125371354618919  0.3265025976530825
  0.2514851814737240  0.0045310479164662  0.6751786525035240
  0.7519257865053077  0.0049408151931374  0.6692111676983127
  0.2493965714651159  0.4991429742722667  0.6684511903144252
  0.7539462587188239  0.4961076904111045  0.6570326486177630
  0.9821291881215718  0.2699992895878083  0.0054312423664487
  0.5118015337288949  0.2658287877715651  0.9997272943580681
  0.9865094306509499  0.7309151825620028  0.9922795056417687
  0.5178964230269143  0.7354590401152700  0.9813497758235737
  0.0055331324071474  0.2486359785966999  0.3194506700312716
  0.4986738774378517  0.2630598951958354  0.3216523809825768
  0.0081958865641134  0.7534164907761152  0.3292210853227828
  0.5006504509532990  0.7325986327260652  0.3256954330226453
  0.9975611690340127  0.2534841904983748  0.6683883595171717
  0.5004629168181415  0.2505658704714866  0.6678510669862371
  0.9985685789305290  0.7460708445666819  0.6642301259183614
  0.4979526722764046  0.7481181201434970  0.6645917403489183


x=0.5
   1.00000000000000
     11.0038653875595660    0.0084267900896755    0.0127169095596167
      0.0098334030867621   11.0257318782003395    0.0408360760744086
      0.0145342549046992    0.0443486259014872   12.1757993852710342
   Cu   Pb   Zr   Ti   O
    12    12     3    21    72
Direct
  0.0028486840904443  0.9992937433928969  0.8397673809556283
  0.0062550529761239  0.9998667831887976  0.1834251692939254
```

| | | |
|---|---|---|
| 0.2507214783715662 | 0.7501869895508707 | 0.8401697692567117 |
| 0.7495638516697073 | 0.2567620967492904 | 0.2047639537709112 |
| 0.4976792899615399 | 0.9985027467366647 | 0.8660432680980673 |
| 0.4947195132778748 | 0.0107491734823023 | 0.1816723193814750 |
| 0.0031788960638548 | 0.4999478589278220 | 0.2000345690148783 |
| 0.2501151029943388 | 0.2466083792423345 | 0.8657714837215886 |
| 0.2467726158268063 | 0.2501525424654937 | 0.1824964368484226 |
| 0.9948256848316436 | 0.9970828944730635 | 0.5254024521141910 |
| 0.2499130778290452 | 0.7469423282266792 | 0.5332222679149576 |
| 0.7471746582134259 | 0.7525149560803644 | 0.5344617409403366 |
| 0.4935991827827536 | 0.5012111100464192 | 0.9681649494453050 |
| 0.4861989929802433 | 0.4925248032744578 | 0.3172919908597349 |
| 0.2232757785422747 | 0.7619722565202797 | 0.2985367352784969 |
| 0.7495379644517070 | 0.2473498997193662 | 0.9640252871276278 |
| 0.9950618247683038 | 0.4943195384944631 | 0.9616534179958455 |
| 0.7401792372344059 | 0.7425493998815178 | 0.9644570911425574 |
| 0.7733142603991395 | 0.7398120499482592 | 0.3038952053124530 |
| 0.4966857229718218 | 0.4982302112158980 | 0.6344392092015572 |
| 0.7548218407564430 | 0.2384802307084564 | 0.6314680361227024 |
| 0.5084014863240779 | 0.9978897656511373 | 0.6285539974097109 |
| 0.9987547723160459 | 0.4919352336233649 | 0.6334652190194263 |
| 0.2498340891550266 | 0.2489105576827221 | 0.6263891543500103 |
| 0.4956579477598369 | 0.7471580075346722 | 0.1436379036847291 |
| 0.7542328810921874 | 0.9983940393714068 | 0.1447315488238700 |
| 0.2516226041928124 | 0.0079632099414492 | 0.4681426066702113 |
| 0.5036945580880485 | 0.2511422820478224 | 0.4647699872032720 |
| 0.2509444780879623 | 0.9979629008527042 | 0.8173431943350038 |
| 0.4966022585227423 | 0.2590186541463033 | 0.1462556675329812 |
| 0.2455652629502236 | 0.9977203408595267 | 0.1297297672175154 |
| 0.7472315227495570 | 0.0084367999565680 | 0.4692337383892735 |
| 0.9990778130209973 | 0.2556545851964208 | 0.4617297889381554 |
| 0.2474251229627467 | 0.4896522352957843 | 0.4733285852383693 |
| 0.5072217073448579 | 0.7436021179563114 | 0.8061517056599596 |
| 0.9912635380669702 | 0.7426519623323489 | 0.8022654849343279 |
| 0.0024790359159385 | 0.2530942543295876 | 0.1500984047394018 |
| 0.2511325196437073 | 0.5053455579443022 | 0.1370012529765161 |
| 0.5062014160046633 | 0.2541384627317012 | 0.8154151532666442 |
| 0.7458655541951956 | 0.5032503171565605 | 0.1518440712788637 |
| 0.0047885905364205 | 0.7479789335004502 | 0.1250232000368233 |
| 0.7515519034162523 | 0.9948603911530566 | 0.8129096001120590 |
| 0.9951200776242307 | 0.2533918256791902 | 0.8125056729667429 |
| 0.5013532583837262 | 0.7465687608466797 | 0.4795937225979000 |
| 0.2483680321536799 | 0.5002682331509287 | 0.8117826401799452 |
| 0.7499458935424788 | 0.4889861775429143 | 0.4746841234043752 |
| 0.9986643657892786 | 0.7433679396154158 | 0.4906517988154004 |
| 0.7497677120288841 | 0.4996565158520425 | 0.8006148978481017 |
| 0.3731294347670349 | 0.1286324515754737 | 0.1642585170031900 |
| 0.8762844945261201 | 0.1362848635513636 | 0.1768219876485567 |
| 0.3772524986189241 | 0.6103957547566239 | 0.1640193893513775 |
| 0.8865123031058912 | 0.6216084065277055 | 0.1737212235718382 |
| 0.3800926008230235 | 0.1343685635971698 | 0.4946979560438916 |
| 0.8781047667542655 | 0.1210680831716150 | 0.5021090466711049 |
| 0.3731570035358625 | 0.6253088260565687 | 0.5097201567311811 |
| 0.8644764204669173 | 0.6320557132735637 | 0.5035012436644973 |
| 0.3780030082921360 | 0.1275604620304097 | 0.8504561987153654 |
| 0.8786496445317199 | 0.1234751370903225 | 0.8298832265005784 |
| 0.3727043015660745 | 0.6251437187459468 | 0.8268667938926826 |
| 0.8754015221899898 | 0.6181610966569345 | 0.8285549228732744 |
| 0.1243508150905981 | 0.3797678971199625 | 0.1725912235488642 |
| 0.6281677045520774 | 0.3781838974028535 | 0.1728977287395812 |
| 0.1252250105188877 | 0.8698449016793445 | 0.1496936067122748 |
| 0.6131736894538974 | 0.8875567132899157 | 0.1783140865899577 |
| 0.1252560773006020 | 0.3727377829322915 | 0.4970385347164092 |
| 0.6210540738756459 | 0.3802128812472181 | 0.4989917917181656 |
| 0.1201462787553996 | 0.8730798913935572 | 0.5206134699880610 |
| 0.6290716591621619 | 0.8724910259576637 | 0.5031790753051379 |

| | | |
|---|---|---|
| 0.1276934139797653 | 0.3705923925791195 | 0.8417266394775815 |
| 0.6282227723740624 | 0.3732640220015054 | 0.8314303975277655 |
| 0.1248251922018958 | 0.8722512633254211 | 0.8265681030772911 |
| 0.6209023382223111 | 0.8779762639066857 | 0.8324199151294700 |
| 0.3659920842155662 | 0.3715419756339873 | 0.1635041017248806 |
| 0.8780622706879448 | 0.3822949114127481 | 0.1851161001342065 |
| 0.3772376632519829 | 0.8900878814758652 | 0.1602524469823081 |
| 0.8867315916704107 | 0.8681296341132591 | 0.1641584543669088 |
| 0.3799564004485996 | 0.3804399972029531 | 0.5003071821508817 |
| 0.8759019051375715 | 0.3756315871898643 | 0.4927257644604712 |
| 0.3706686369932894 | 0.8645052887327939 | 0.5062548303391577 |
| 0.8720187844715841 | 0.8761023979165256 | 0.5142901566137159 |
| 0.3721336279072650 | 0.3698865219628785 | 0.8405835965707101 |
| 0.8737723544268543 | 0.3745213324021939 | 0.8340881286884575 |
| 0.3761976636386801 | 0.8725041634427403 | 0.8362198212612614 |
| 0.8812895522290300 | 0.8788915395238536 | 0.8213041766322841 |
| 0.1258173335964725 | 0.1291043603401490 | 0.1582891841209580 |
| 0.6182247832553979 | 0.1316726393156817 | 0.1768959520873550 |
| 0.1213842890845460 | 0.6213396516418872 | 0.1680008175593707 |
| 0.6480970917013729 | 0.6388383932856296 | 0.1625721352934684 |
| 0.1108612850079781 | 0.1229470278123304 | 0.5093598338177703 |
| 0.6275270492192532 | 0.1286100080478892 | 0.4920640845236094 |
| 0.1324478346223096 | 0.6279562391841738 | 0.5075653501290186 |
| 0.6272826656839114 | 0.6281737828064746 | 0.5107213753650939 |
| 0.1231579598425744 | 0.1262830962951201 | 0.8375162076481476 |
| 0.6191464367866077 | 0.1223279780584554 | 0.8424646189374843 |
| 0.1292640635329518 | 0.6274358539566812 | 0.8273337666099403 |
| 0.6268599244188706 | 0.6195068607021934 | 0.8238308534805695 |
| 0.2467570717624733 | 0.0005458422026582 | 0.9894569201162857 |
| 0.7539878766118741 | 0.9975304557434783 | 0.9846086604760333 |
| 0.2504130386136857 | 0.5038564873151072 | 0.9936238432062551 |
| 0.7550301124095374 | 0.4989496198192568 | 0.0040697974097152 |
| 0.2317106673651851 | 0.9811211160814530 | 0.3136732857895277 |
| 0.7612858226946556 | 0.9885429087638434 | 0.3301533684624681 |
| 0.2598918270222784 | 0.5058750304212319 | 0.3314253923326955 |
| 0.7402234321033878 | 0.5157968592265878 | 0.3314798221362986 |
| 0.2665967894404013 | 0.0173300965993039 | 0.6744364338982571 |
| 0.7331644770663742 | 0.0170285265525822 | 0.6684571603787318 |
| 0.2486317149973939 | 0.4829931201187890 | 0.6677826862276793 |
| 0.7552020803526628 | 0.4878005314119126 | 0.6577939790534116 |
| 0.9845212509640330 | 0.2670446849529713 | 0.0054961449072402 |
| 0.5160126843857962 | 0.2664748777858753 | 0.0031625146802839 |
| 0.9906178132466650 | 0.7362538299260979 | 0.9853054130974838 |
| 0.5166714080779458 | 0.7336172829093415 | 0.9831598644592365 |
| 0.0071417579397523 | 0.2474850365198374 | 0.3232252956528184 |
| 0.4971871200228588 | 0.2672576902512386 | 0.3234247789204797 |
| 0.0078310424345733 | 0.7538119629526520 | 0.3448214364788110 |
| 0.4984743656385071 | 0.7356552727445220 | 0.3349722321505275 |
| 0.9994695894875479 | 0.2662977568663019 | 0.6689116136800216 |
| 0.5002290938674459 | 0.2475647794887808 | 0.6699185979183433 |
| 0.9953182343834772 | 0.7328013928174061 | 0.6613618025522113 |
| 0.5025294647531150 | 0.7535506769261059 | 0.6631693748920603 |
